# Zn Subcellular Distribution in Liver of Goldfish (*Carassius Auratus*) with Exposure to Zinc Oxide Nanoparticles and Mechanism of Hepatic Detoxification

**DOI:** 10.1371/journal.pone.0078123

**Published:** 2013-11-01

**Authors:** Wenhong Fan, Qian Li, Xiuping Yang, Li Zhang

**Affiliations:** 1 Department of Environmental Science and Engineering, School of Chemistry and Environment, Beihang University, Beijing, China; 2 Key Laboratory of Marine Bio-resources Sustainable Utilization, South China Sea Institute of Oceanology, Chinese Academy of Sciences, Guangzhou, China; The Ohio State University, United States of America

## Abstract

Zinc Oxide Nanoparticles (ZnO NPs) have attracted increasing concerns because of their widespread use and toxic potential. In this study, Zn accumulations in different tissues (gills, liver, muscle, and gut) of goldfish (*Carassius auratus*) after exposure to ZnO NPs were studied in comparison with bulk ZnO and Zn^2+^. And the technique of subcellular partitioning was firstly used on the liver of goldfish to study the hepatic accumulation of ZnO NPs. The results showed that at sublethal Zn concentration (2 mg/L), bioaccumulation in goldfish was tissue-specific and dependent on the exposure materials. Compared with Zn^2+^, the particles of bulk ZnO and the ZnO NPs appeared to aggregate in the environmentally contacted tissues (gills and gut), rather than transport to the internal tissues (liver and muscle). The subcellular distributions of liver differed for the three exposure treatments. After ZnO NPs exposure, Zn percentage in metal-rich granule (MRG) increased significantly, and after Zn^2+^ exposure, it increased significantly in the organelles. Metallothionein-like proteins (MTLP) were the main target for Zn^2+^, while MRG played dominant role for ZnO NPs. The different results of subcellular distributions revealed that metal detoxification mechanisms of liver for ZnO NPs, bulk ZnO, and Zn^2+^ were different. Overall, subcellular partitioning provided an interesting start to better understanding of the toxicity of nano- and conventional materials.

## Introduction

In recent years, following the mass production and widespread use of nanomaterials, their toxic effects have attracted increased concerns because of their substantially different properties from traditional materials [1], [2]. Zinc Oxide Nanoparticles (ZnO NPs), one of the most widely used nanomaterials, are already produced in industrial scale [3], [4]. It will certainly be used more widely in different industries as a result of their unique advantages in industrial and medicinal products. Direct and indirect releases of nanoparticles (NPs) into aquatic environments via engineering applications and sewage effluent will also increase the exposure of humans and ecosystems to NPs.

Consequently, the potential effects of ZnO NPs on aquatic ecosystems have attracted special attentions [5], [6]. ZnO NPs have been investigated in various aspects, including biochemistry and toxicology [7], [8], and were confirmed to be one of the most harmful NPs in aquatic environment [9]. Although some disagreements exist, the generally accepted toxicity mechanisms of ZnO NPs include particle effects and dissolved free-ion effects [10]. The solubility of ZnO NPs (in the milligram-per-liter range) was similar to that of bulk ZnO, and at concentration of 60 µg Zn/L, the 72 h IC 50 to a freshwater alga (*Pseudokirchneriella subcapitat*a) was similar with the bulk ZnO [11]. But to the bacteria, ZnO NPs showed higher toxicity than their bulk counterparts [12]. In general, the environmental behavior and biological effects of ZnO NPs are complicated and unpredictable.

A common approach for the study of toxic effects of metal oxide NPs is the metal accumulation on different species [13]. But as one of the essential elements, Zn accumulated in the organisms does not inevitably lead to toxic effects. The toxicity of metals only occurs when the cellular concentration reaches a critical level and the intracellular homeostasis is disordered. The detoxification mechanisms of heavy metals in organisms are also complicated. An important defensive response for organisms to metal challenge is storing these metals by sequestration and then detoxification [14], [15], such as binding with metallothioneins (MT) and metal-rich granule (MRG) [16], [17].

Subcellular distribution of metals in organism may reflect the internal processing of metal accumulation and provide valuable information about metal toxicity and tolerance. For life-essential metals, such as Zn, the balance between essentiality and toxicity was regulated through the binding to specific cellular sites, and the knowledge of intracellular localization became crucial [18]. Furthermore, whether ZnO NPs could enter into the internal tissue in the form of particles or not was still controversial. The information of intracellular Zn distribution might provide a better understanding of the toxicity of ZnO NPs. However, the technique of subcellular metal partitioning has not been applied to investigate the effects of different kinds of metal materials (ZnO NPs, bulk ZnO, and Zn^2+^).

The main objective of this study is to investigate whether the fish toxicity of ZnO NPs was size- or composition- related by comparing the NPs to their bulk counterparts. The accumulation level of different Zn materials in different tissues (gills, liver, muscle, and gut) is tested. Furthermore, the technique of metal subcellular distribution is used for the first time to investigate the toxicity mechanism of ZnO NPs in the liver of goldfish (*Carassius auratus*). Goldfish are commonly existed in freshwater of China and are widely used in toxic test. Our aim is to understand the effects and possible mechanisms of ZnO NPs on goldfish in order to provide more information on their toxicological effects in aquatic ecosystem.

## Methods

### Ethics Statement

This study was carried out in strict accordance with the recommendations of the approval of Institutional Authority for Laboratory Animal Care of Beihang University. All efforts were made to minimize suffering of the fish.

### Preparing NPs Suspensions

The ZnO NPs were provided by Nanjing High Technology Material Co., Ltd. The surface area of the NPs was measured using a Nova 2200e Brunauer-Emmett-Teller (BET) surface area analyzer (Quantachrome, Boynton Beach, FL). The ZnO NPs and bulk ZnO stock suspensions (100 mg/L) were prepared as follows: 100 mg ZnO NPs or bulk ZnO particles were transferred to 1 L ultra-pure water (pH 5.7, filtered through a 0.2 µm sieve) (Barnstead, EASYpure II, USA) and dispersed using a ultrasonic cleaner for at least 30 min to break them into small particles. The stock solutions were stored at room temperature without addition of salts or dispersants. The images of the ZnO NPs and bulk ZnO in ultrapure water were characterized using transmission electron microscopy (TEM) (JEOL, JEM-2100F), operated at 100 kV acceleration voltage. Droplets of solution were injected onto a cleaned 200 mesh copper carbon grids using a capillary tube. After drying for one day at room temperature, the samples were then placed in the TEM for imaging.

Ionic zinc stock solution (Zn^2+^) was prepared with ZnCl_2_ identically to the ZnO NPs and bulk ZnO, but was not sonicated. The stock solutions were stored at room temperature before diluting to exposure concentrations.

### Model Organism

Goldfish (*Carassius auratus*), ranging from 20 g to 30 g in weight and from 12 cm to 15 cm in total length, were obtained from the Shi Li He Flower and Fish Market in Beijing. Prior to the experiments, the fish were cultured in the laboratory at room temperature with a light:dark cycle of 16∶8 h for more than one week before experiments. The water used for the acclimation period was tap-water, which was aerated for 3–5 days to eliminate residual chlorine. The fish were not fed during the acclimation period or the exposure experiments to avoid the influence of food.

### Exposure Experiments

The stock solutions of ZnO NPs, bulk ZnO, and Zn^2+^ were prepared as described before. The toxicity-test concentration of these three materials was set to the same Zn concentration of 2 mg/L. The water used for exposure experiments was the same as acclimation period. The water quality was controlled as follows: pH 6.8–7.2, dissolved oxygen concentration 100% saturation (aerated), temperature 23–25°C, dissolved organic carbon (DOC) concentration 1.04 mg/L, hardness of CaCO_3_ 200 mg/L, conductivity 640 µS/cm. After dispersed using a sonicator for 30 min, 200 ml stock solutions were add into 9.8 L pretreated-tap-water to obtain the expectant exposure concentration (2 mg/L). The exposure experiment was conducted at room temperature with a light:dark cycle of 16∶8 h. 10 fish with similar body weights were added in each container for 30-day exposure. One control sample with no added Zn was maintained under the same conditions. For each exposure conditions and the control, three replicates were used. The exposure media were renewed every 5-day and the fish were not fed during the exposure experiments.

After the 30-day exposure, the mortality was observed. After that, two fish were removed from each container and depurated in pretreated-tap-water for 10 min. One fish was used for test of Zn accumulation. The fish were weighed and dissected, and the tissues (gills, liver, muscle, and gut) were collected. The other fish was used for test of Zn subcellular distribution. The fish were weighed and only liver was collected.

### Zn Concentration in Different Tissues after Exposure

A portion of the tissues (about 0.1 g) collected before was dried at 80°C to constant weight and then digested in 68% HNO_3_ (2 ml, 68%, GR) at 120°C until the digestion solution was clear and transparent. During digestion, 0.2 ml hydrogen peroxide (H_2_O_2_, 30%, GR) was added to ensure complete digestion. After cooling, all solutions, including the washing solution were transferred to a volumetric flask and diluted. Concentration of Zn was analyzed with Inductively Coupled Plasma-Mass Spectrometry (ICP-MS, VG PQ2 TURBO). The Zn accumulation was calculated based on the dry weight of the tissues (µg/g dry weight).

Yttrium was used as the internal standard. Procedural and reagent blanks were analyzed for quality control. Standard Reference Material (mussel tissue, SRM 2976, National Institute of Standards and Technology, USA) was used and analyzed for Zn in the same manner as all of the experimental samples, and the recovery ratio of Zn ranged from 94.6% to 103.2%.

### Subcellular Distribution in the Liver

**Figure 1:**
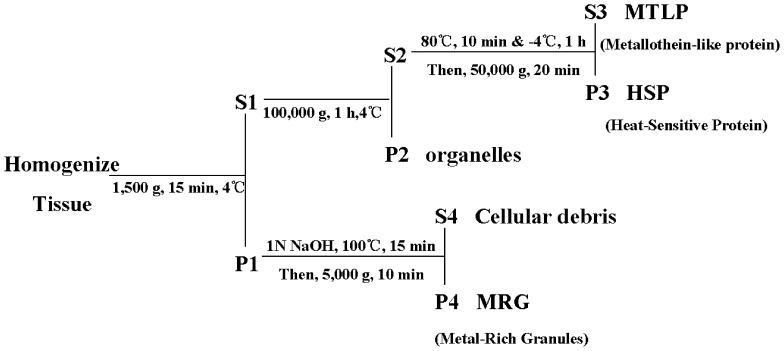
Procedure for determining the subcellular partitioning of metal. The liver was homogenized by different treatments and centrifuged at different speeds, afterwards the following subcellular fractionations were obtained: organelles (i.e. nucleus, mitochondria, and microsomes, P2), HSP (heat-sensitive proteins, P3), MTLP (metallothionein-like protein, S3), MRG (metal-rich granules, P4), and cellular debris (S4).

The liver of fish collected was homogenized and centrifuged according to a procedure modified from that employed by Wallace et al. [17] (Fig. 1). Simple description was as follows:

Liver from individual goldfish was transferred into a pre-weighed polypropylene tube (10 ml). Wet weights were obtained for individual goldfish. Liver was then homogenized with a tissue homogenizer for 30 s on medium speed in 3 ml (∼1∶10 tissue to buffer ratio) of cold sucrose buffer (0.25 mol/L sucrose, 0.1 mol/L Tris-HCl, pH 8.6). After that, homogenized tissue was first fractionated by centrifugation at 1,500×g (15 min, 4°C), producing a pellet (P1) containing tissue fragments and other cellular debris (e.g. membranes, MRG) and a supernatant (S1) containing cytosol. S1 was centrifuged at 100,000×g (1 h, 4°C) to produce a pellet (P2) containing various organelles (i.e. nucleus, mitochondria and microsomes). S2 contained cytosol. The cytosol was then heat-denatured (80°C, 10 min, followed by ice-bath, 1 h) and centrifuged at 50,000×g (4°C, 20 min). This final pellet (P3) contained heat-sensitive proteins (HSP, e.g. enzymes), while the supernatant (S3) contained metallothionein-like protein (MTLP). Metal-rich granules (MRG) were isolated from P1 through digestion of tissue fragments with NaOH (1N, 100°C, 15 min) and centrifugation at 5,000×g (10 min). The resulting supernatant (S4) contained metal associated with dissolved tissues.

Finally, the following subcellular fractionations were obtained: organelles (P2), HSP (P3), MTLP (S3), MRG (P4), and cellular debris (S4). They were digested with HNO_3_ (68%, GR) and then diluted to an appropriate volume. The Zn concentration was measured by ICP-MS (VG PQ2 TURBO).

### Statistical Analysis

The data were presented as means ± standard deviation (SD). The Zn accumulation and subcellular distribution data were analyzed using analysis of variance [(ANOVA), SPSS Statistics 19.0.1, IBM]. The normality and homogeneity of variances were tested when performing the analysis of variance. The percentage data were arcsine-transformed before introduction to ANOVA. Mean values were considered different at *p*<0.05.

## Results and Discussion

### Characteristics of ZnO NPs and Bulk ZnO

**Figure 2:**
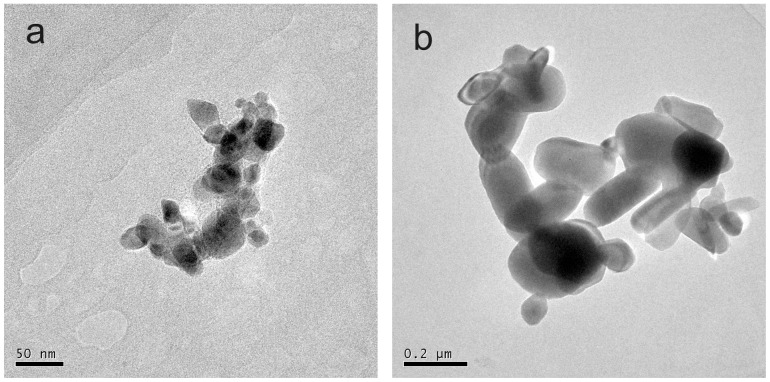
TEM images of ZnO suspensions in ultrapure water: (a) ZnO NPs and (b) bulk ZnO.

The ZnO NPs (purity>99%) used in this study were single crystal without any surface modification. The surface area for ZnO NPs and bulk ZnO powder estimated by BET were 96.31 m^2^/g and 3.33 m^2^/g, respectively. The TEM images of the ZnO NPs and bulk ZnO in ultrapure water were shown in Fig. 2. The initial diameter of the individual ZnO NPs (approximately 30 nm) was much smaller than the bulk ZnO particles (approximately 0.2 µm). The ZnO NPs were spherical and existed as small agglomerates (50–150 nm). The particles of bulk ZnO were ellipsoidal, and the aggregate size was much larger than ZnO NPs.

Zn^2+^ concentrations measured in the exposure water were 0.75±0.08 mg/L, 0.68±0.11 mg/L, and 1.89±0.09 mg/L for ZnO NPs, bulk ZnO, and Zn^2+^, respectively. The solubilities of ZnO NPs and bulk ZnO in the exposure water were close which was approximately 30∼35%, indicating that a portion of ZnO NPs and bulk ZnO existed in the form of particles. The solubilities were a little higher than the results obtained by Franklin et al. [11], who studied the dissolution rates of bulk ZnO and ZnO NPs of nominal total Zn concentration 100 mg/L. That might be due to the conditions of the exposure water.

### Zn Accumulation in Tissues

**Figure 3:**
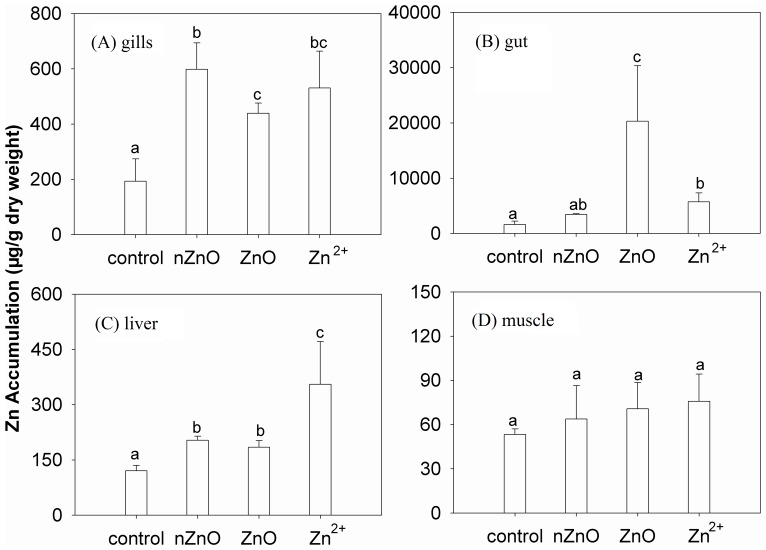
Zn accumulations in (A) gills, (B) gut, C) liver, and (D) muscle after 30-day exposure with ZnO NPs (nZnO), bulk ZnO (ZnO), and Zn^2+^. Values are means ± SD (*n* = 3). Bars with different letters (a, b, c) show the significant difference (*p*<0.05).

During the 30 day of exposure, no deaths occurred. The Zn accumulations in different tissues of the goldfish exposed to ZnO NPs, bulk ZnO, and Zn^2+^ after 30 days were shown in Fig. 3. Compared with the control, exposure to ZnO NPs resulted in significant Zn accumulations in the gills and liver (*p*<0.05), while exposure to bulk ZnO and Zn^2+^ both resulted in significant zinc accumulations in the gills, gut, and liver (*p*<0.05). Zn concentrations in different tissues were different, with the following sequence: gut>gills>liver>muscle. Overall, the tissue accumulations were similar to other literatures. Gut and gills were the crucial Zn accumulation tissues, liver came the second, and Zn contents in muscle were the least [19], [20].

Zn is an essential metal for goldfish as co-factor of many metallo-organic complexes [21]. The Zn exposure regimes employed in the present study were sublethal for goldfish as no death was observed during the experiment. However, environmental zinc disturbed the internal Zn homeostasis. A fundamental concept of toxicology is that adverse effects occur when toxic agents accumulate at target sites in adequate concentrations and for adequate time [18]. The results of Zn accumulation in different tissues of goldfish exposed to ZnO NPs, bulk ZnO, and Zn^2+^ showed that accumulation was tissue-specific and different among the exposure treatments.

Gills and gut are two tissues to take up metals from water and food, and the absorbed metals are transferred to other internal tissues (e.g. liver and muscle) via diffusion and blood circulation [22]. The present study demonstrated that compared with Zn^2+^, the bulk ZnO and the ZnO NPs appeared to aggregate Zn in the environmentally contacted tissues (gills and gut), and the internal tissues (liver) had a somewhat lower risk.

In the gills [Fig. 3(A)], Zn contents of three exposure treatments were all over twice of that with the control, and the accumulations were statistically different after exposure to ZnO NPs and bulk ZnO (*p*<0.05). The gills have been well investigated as the dominant Zn^2+^ uptake site in freshwater fish by previous studies [23]. It had been reported that gills of *Salmo gairdneri* filtered a large amount of water for respiratory gas exchange [24]. In the meantime, fish had the opportunity to absorb waterborne metals [25]. Zn^2+^ had been well known, at least partially, to be taken up by the gills via apical Ca channels and base lateral Ca-ATPase located in the mitochondrial-rich, ion-transporting chloride cells [26]. As described above, the concentrations of Zn ions in solutions of ZnO NPs and bulk ZnO were much lower than that in Zn^2+^ solution. But Zn accumulation in gills for the bulk ZnO treatment was not different from the Zn^2+^, and for ZnO NPs it was even higher. It demonstrated that a part of Zn accumulated in gills of goldfish treated with ZnO NPs and bulk ZnO was due to the aggregation of undissolved particles.

In the gut [Fig. 3(B)], the Zn accumulation after exposure to ZnO NPs was not statistically different from the control, but for bulk ZnO, it was much higher than those after exposure to ZnO NPs and Zn^2+^. Intestinal Zn uptake was considered as involving the Ca channel and replacement of K^+^ with Na^+^ in the medium stimulated uptake of Zn at high Zn (II) concentration [27]. The unusual Zn accumulation after exposure to bulk ZnO was likely due to the aggregation of large particles on the surface of intestinal villi [28]. Interestingly, compared to bulk ZnO and Zn^2+^, Zn accumulation in the gut was the least for the ZnO NPs exposure. The reason was not clear yet. It may because of the low concentration of Zn^2+^ ions in ZnO NPs solution, and the much smaller aquatic particle size of ZnO NPs compared with that of the bulk ZnO.

In the liver [Fig. 3(C)], the Zn accumulations after all the treatments were statistically different from the control (*p*<0.05). Meanwhile, after exposure to Zn^2+^, Zn accumulation was obviously higher than that for the nano or bulk ZnO (*p*<0.05). Liver had been recognized as a specialized tissue for metal metabolism and detoxification [29]. The detoxification ability of the liver was due to the activity of MTs and MTLPs [30]. The relatively low accumulations of Zn after exposure to ZnO were likely due to the limited solubility of the bulk and nano ZnO [31]. The results also suggested that the extremely high Zn accumulation in the gut after the bulk ZnO exposure was unlikely to be transported to the internal tissue of fish.

In the muscle tissue [Fig. 3(D)], however, the Zn concentration did not change obviously in any exposure treatments. The metal accumulation capacity of the fish muscle has been known as lower than that of the other tissues and organs such as skin, gills, gut, and liver. It was possibly because of the less metal-binding protein (e.g., MT) in the muscle [32], [33]. Furthermore, because of the large muscle mass, Zn concentration in the muscle was usually hard to elevate by Zn exposure in most cases [34].

### Zn Subcellular Partitioning in the Liver and Mechanism of Hepatic Detoxification

Liver is a tissue of metal metabolism and detoxification. In this study, technique of subcellular partitioning is used for the first time on the liver of goldfish, to study the mechanism of hepatic detoxification of Zn after exposure to ZnO NPs, bulk ZnO and Zn^2+^. It had been reported that in gills, the detoxification and metal-sensitive fractions occurred concurrently with no evidence of selective protection [18]. We wondered whether the liver, the dominating tissue of detoxification, had the function of selective protection on the metal-sensitive fractions.

**Figure 4:**
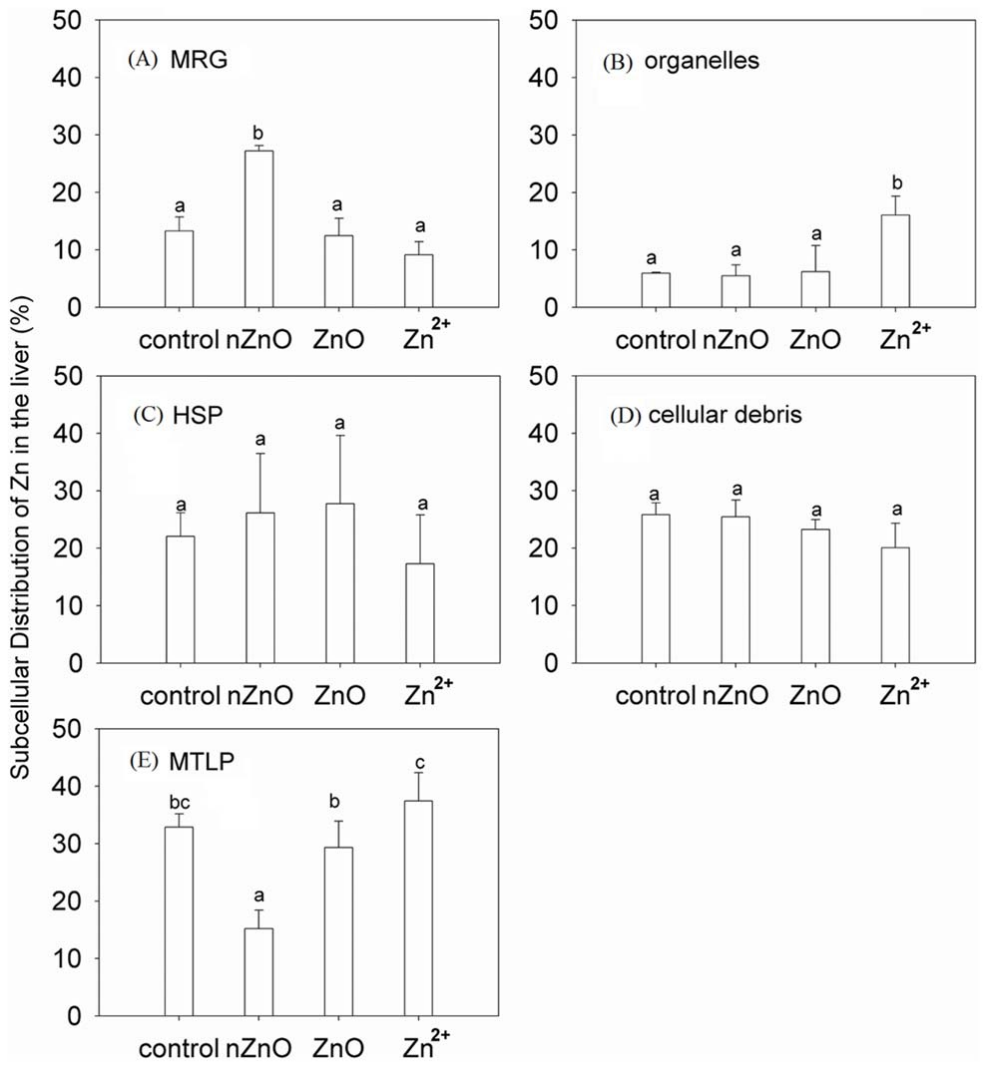
Subcellular Zn distribution (%) in (A) MRG, (B) organelles, (C) HSP, (D) cellular debris, and (E) MTLP of the liver after 30-day exposure with ZnO NPs, bulk ZnO, and Zn^2+^. Values are means ± SD (n = 3). Bars with different letters (a, b, c) show the significant difference (*p*<0.05).

The percentages of subcellular Zn distributions in the liver after the 30-day exposure to ZnO NPs, bulk ZnO, and Zn^2+^ were shown in Fig. 4. In the control, the MTLP was the main binding site of Zn (32.88%). After the ZnO NPs exposure, the percent of Zn increased obviously in MRG but decreased in MTLP (*p*<0.05). After the bulk ZnO exposure, Zn distributions (%) were not change obviously in any subcellular portion. After the Zn^2+^ exposure, the percentage of Zn in the organelles increased significantly (*p*<0.05), whereas those in other subcellular portions did not change obviously.

In general, the subcellular Zn distribution for ZnO NPs was significantly different from Zn^2+^ exposure in MRG, organelles, and MTLP, revealing the different mechanism of hepatic Zn accumulation for these two materials. In the control and Zn^2+^ exposure treatment, the MTLP fraction was the dominant Zn binding position. After Zn^2+^ exposure, goldfish sequestered more than 30% of its total hepatic Zn in MTLP. Most of the previous studies attributed the hepatic detoxification function to the MTLP synthesis [35]. The role of MTLP in binding with heavy metals was due to its chemical nature of abundant disulfide bonds. However, it was different from the results obtained by Goto et al. [36], who observed the whole body Zn subcellular partitioning for mummichogs, and proved the importance of MRG in metal deposition. It had been clarified that metal detoxification could also take place through the precipitation of metals into insoluble concretions [17]. But it was difficult to assess the relative importance of MRG and scale deposition from Goto’s study, as the results were based on the whole body of fish. In the present study, liver was an internal tissue, and the detected Zn was regarded as intracellular. An interesting observation was the relatively high proportion in MRG and low proportion in MTLP of hepatic Zn after exposure to ZnO NPs, which perhaps a reflection of differences in detoxification strategies. As MRG could precipitate some of the hazardous particles in the cells, it was presumed that a small quantity of ZnO NPs penetrated into the hepatic cells via blood or interstitial space in the form of nanoparticles. The size of the NPs, which fell in the transitional zone between individual atoms or molecules and the corresponding bulk materials, created the opportunity for increased uptake and interaction with biological tissues [37].

In previous studies, MTLP and MRG were considered in tandem as a compartment and defined as biologically detoxified metal (BDM) [17]. In consideration of the similar role of MRG and MTLP on metal detoxification, a large portion of hepatic Zn after all the exposure treatments was of no potential mechanisms of toxicity. It stated that the liver of goldfish had a priority to accumulate the metals in BDM.

However, the detoxify function of MTLP and MRG was not all-purpose. After treatment of Zn^2+^ exposure, the percentage of Zn in the organelles (e.g. nuclei, mitochondria, microsomes, and lysosomes) increased significantly. Wang et al. [38] pointed out that the Zn bound to the organelles, which was probably trapped in the lysosomes (membrane-bound cell organelles containing hydrolytic enzymes), might have a significant impact on the physiological functions of cells. Giguère et al. studied subcellular metal distributions in indigenous yellow perch (*Perca flavescens*), and observed Zn accumulation in all kinds of organelles (mitochondria, microsomes, and lysosomes) [39]. In this study, organelles were considered as a single component which was sensitive to metal toxicity. So it is not discussed a lot here since the concerns were focused more on the amount of the metal distributed to the different subcellular fractions (metal-sensitive or metal-detoxified) and the metal redistribution among those fractions than the physiological functions of the metals.

After treatment of Zn^2+^ exposure, Zn proportion in HSP showed little changes while that in organelles was more than doubled, indicating that organelles were more vulnerable than enzymes. For the treatments of ZnO NPs and bulk ZnO, Zn proportions in organelles and HSP were not changed obviously, showing little effects on the metal-sensitive fractions (HSP and organelles). It was speculated that the differences were mainly due to the difference of total hepatic Zn accumulations. Zn regulation in fish exposed to variable ambient Zn concentrations had been observed before and reflected the essential nature of zinc and a degree of homeostatic control of intracellular zinc [40]. Zn accumulation after Zn^2+^ exposure was beyond the capability of liver to maintain homeostasis. On the whole, toxicity of ZnO NPs on liver of goldfish was not as obvious as Zn^2+^ because of its limited solubility and the detoxification of MRG.

Overall, subcellular localization provided an interesting start to get better understanding of the toxicity of nano- and conventional materials. More work is needed to disclose the specific composition and the detoxication role of each subcellular component.

## Conclusion

It was found that at the sublethal concentration of Zn, the bioaccumulations of goldfish after exposure to ZnO NPs, bulk ZnO, and Zn^2+^ were tissue-specific and dependent on the exposure. In addition, compared with Zn^2+^, the bulk ZnO and the ZnO NPs appeared to aggregate Zn in the environmentally contacted tissues (gills and gut), rather than transport Zn to the internal tissues (liver and muscle). In addition, the difference of subcellular partitioning between MTLP and MRG indicated that the mechanism of metal detoxification of liver for ZnO NPs and Zn^2+^ was different in a way. Metallothionein-like proteins (MTLP) were the main target for Zn^2+^, while MRG played the dominant role for ZnO NPs. Overall, the results indicated that subcellular partitioning could be an interesting start to get more understanding of the toxicity of nano- and conventional materials.
